# Rolling Back a Malaria Epidemic in South Africa

**DOI:** 10.1371/journal.pmed.0020368

**Published:** 2005-10-04

**Authors:** Patrick E Duffy, Theonest K Mutabingwa

## Abstract

The authors discuss the success in malaria control in KwaZulu-Natal (reported by Barnes and colleagues), and its implications for the rest of Africa.

A mid the dire statistics showing a deadly resurgence of malaria, a notable success has been scored in South Africa. In KwaZulu–Natal province, malaria cases increased from about 600 in 1991–1992 to more than 30,000 by 1999–2000 [1]. Then, after household spraying with DDT was implemented, and the new antimalarial combination artemether-lumefantrine (AL) was widely deployed (Figure 1), cases declined by more than 99% over the next three years. A paper in *PLoS Medicine* by Barnes et al. [2] examines the implementation and efficacy of AL during the KwaZulu–Natal crisis. They conclude that vector control and widespread use of artemisinin-based combination therapy (ACT) such as AL may confer similar benefits in other African countries. Could the adoption of these policies salvage the Roll Back Malaria Initiative that was formed in 1999 to halve malaria deaths by 2010 [3], but which was recently lamented as “dysfunctional” for its inaction in the face of rising malaria morbidity and mortality rates [4]?

**Figure 1:**
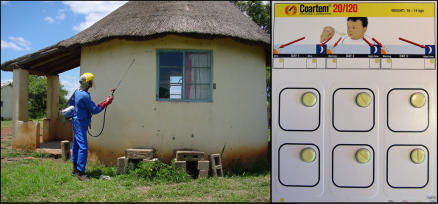
Mosquito Control and ACT Are Both Likely Contributors to the Reduction of Malaria in KwaZulu–Natal (Photo: Karen Barnes and Atis Muelenbachs)

## Artemisinins to the Rescue

Artemisinin derivatives such as artemether have several advantages—they act rapidly, cause few side effects, and have not yet acquired resistant parasites [5]. Artemisinins also prevent parasite transmission by inactivating or killing gametocytes [6]. In northwestern Thailand, malaria incidence declined after the ACT artesunate-mefloquine was introduced, and its effectiveness has been sustained over several years, possibly due to gametocytocidal effects [7]. In a study recently published in *PLoS Medicine* [6], gametocytes from Gambian children treated with AL were less likely than those from children treated with a chloroquine and sulfadoxine-pyrimethamine (SP) combination to infect mosquitoes. These reports have led many to expect that ACTs will dramatically improve case management, and reduce malaria transmission in Africa.

## KwaZulu–Natal—A Special Case?

Caution is warranted, however. KwaZulu–Natal is more similar to Thailand than to most sub-Saharan countries in ways that may affect the influence of ACTs. The economic strength of South Africa supported effective vector control measures and a health-care infrastructure that facilitated prompt diagnosis and treatment. Poorer sub-Saharan countries are unable to support similar programs in the absence of additional financial resources.

Noneconomic issues may also limit the effect of ACTs on malaria incidence and case management in Africa. Malaria transmission and, therefore, immunity are low in both Thailand and KwaZulu–Natal. Thais and South Africans typically get sick when they are infected, and seek treatment. In African countries where malaria transmission is high, semi-immune individuals often do not feel sick enough to seek treatment, and act as a reservoir for continued transmission. Additionally, African children often present with high-density parasitemia, making it more likely that parasites will be temporarily suppressed but then recrudesce after artemisinin therapy [8,9].

ACTs will improve treatment outcomes in areas of sub-Saharan Africa where resistant parasites have rendered the current first-line drugs nearly useless. Drug resistance has probably played a key role in the rising malaria mortality rates among African children [10], so ministries of health are optimistic that ACTs will reverse this awful trend. However, the long-term effectiveness of ACTs in high endemicity areas has not been proven, and many operational questions remain unanswered.

## ACT Alternatives

Are ACTs the most effective new antimalarial combination? In the July issue of *PLoS Medicine*, Dorsey and colleagues reported that the nonartemisinin combination of SP and amodiaquine (AQ) was as effective or better (and cheaper) than the combination of artesunate and AQ for treating Ugandan children, when both recrudescent and new infections were considered [11]. Resistance to both SP and AQ is spreading in Africa, and this will limit the sustainability of the combination. Furthermore, because recrudescent parasites are more likely to be drug-resistant [12], and recrudescences were more common after SP-AQ, this combination may accelerate the spread of resistant parasites. Nevertheless, the combination should be considered as a short-term strategy in areas where the parasite remains sensitive. The results also caution that the benefits of ACTs may be limited in high endemicity areas unless reinfections are promptly treated.

## Is AL the Best ACT for Africa?

AL is the only co-formulated ACT, which improves compliance. Furthermore, a dramatic rollback of malaria has been achieved in KwaZulu–Natal where AL was deployed. These have been strong factors in the selection of AL as first-line therapy by many African countries. However, the sharp decline in malaria in KwaZulu–Natal commenced after DDT spraying of households was initiated and before AL was deployed; therefore, the relative contribution of AL remains unclear. Sustained success with ACTs in Thailand has been achieved with artemether-mefloquine. The long-term effectiveness of AL remains unproven. The extended half-life of lumefantrine and the short half-life of artesunate mean that many reinfections in Africa will be exposed to lumefantrine alone, increasing the odds that resistant parasites will be selected. Worrying reports from Zanzibar suggest that lumefantrine resistance may already be emerging there, not long after AL was introduced as second-line therapy [13]. Future studies should compare different artemisinin and nonartemisinin combination therapies for their long-term effectiveness. Finally, the huge new market for AL in Africa has outstripped the available supplies, delaying the launch of ACTs in some countries, and it remains uncertain when these supply problems will be fully resolved.

## Learning from Success

Will ACTs “roll back malaria”? The Barnes et al. paper focuses on the efficacy and implementation of AL in KwaZulu–Natal, but active surveillance and treatment for asymptomatic carriers [1], as well as residual spraying, contributed to the success. Barnes et al. argue for an effective vector control program and ACT implementation in the context of a well-developed rural primary health-care infrastructure. While the cost of executing these programs throughout Africa may seem great, the cost of not doing so is likely to be greater, especially if resistance to ACTs emerges as a consequence. In any case, the widespread expectation that ACTs alone will turn the tide in the fight against malaria may be unrealistic.

## Abbreviations

ACT: artemisinin-based combination therapy
AL: artemether-lumefantrine
AQ: amodiaquine
SP: sulfadoxine-pyrimethamine
